# Loosening the Lid on Shoulder Osteoarthritis: How the Transcriptome and Metabolic Syndrome Correlate with End-Stage Disease

**DOI:** 10.3390/ijms26073145

**Published:** 2025-03-28

**Authors:** Samuel J. Lynskey, Zihui Ling, Mark Ziemann, Stephen D. Gill, Sean L. McGee, Richard S. Page

**Affiliations:** 1 Department of Orthopaedic Surgery, Geelong University Hospital, Geelong, VIC 3220, Australia; 2 School of Medicine, Faculty of Health, Deakin University, Waurn Ponds, Geelong, VIC 3220, Australia; 3 Peninsula Health, 2 Hastings Rd, Frankston, VIC 3199, Australia; 4 Burnet Institute, Melbourne, VIC 3004, Australia; 5 School of Life and Environmental Sciences, Deakin University, Waurn Ponds, Geelong, VIC 3216, Australia; 6 Barwon Centre for Orthopaedic Research and Education (BCORE), St. John of God Hospital, Geelong, VIC 3220, Australia; 7 IMPACT—The Institute for Mental and Physical Health and Clinical Translation, Barwon Health, Deakin University, Geelong, VIC 3220, Australia

**Keywords:** transcriptomic analysis, metabolic syndrome, OA, cuff-tear arthropathy, rotator cuff arthropathy, ageing

## Abstract

Metabolic syndrome (MetS) associated with Osteoarthritis (OA) is an increasingly recognised entity. Whilst the degenerative pattern in cuff-tear arthropathy (CTA) has been well documented, the biological processes behind primary shoulder OA and CTA remain less understood. This study investigates transcriptomic differences in these conditions, alongside the impact of MetS in patients undergoing total shoulder replacement. In a multi-centre study, 20 OA patients undergoing total shoulder replacement were included based on specific treatment indications for OA and cuff-tear arthropathy as well as 25 patients undergoing rotator cuff repair (RCR) as a comparator group. Tissues from subchondral bone, capsule (OA and RCR), and synovium were biopsied, and RNA sequencing was performed using Illumina platforms. Differential gene expression was conducted using DESeq2, adjusting for demographic factors, followed by pathway enrichment using the mitch package. Gene expressions in CTA and primary OA was differentially affected. CTA showed mitochondrial dysfunction, *GATD3A* downregulation, and increased cartilage degradation, while primary OA was marked by upregulated inflammatory and catabolic pathways. The effect of MetS on these pathologies was further shown. MetS further disrupted WNT/β-catenin signalling in CTA, and in OA. Genes such as *ACAN*, *PANX3*, *CLU*, and *VAT1L* were upregulated, highlighting potential biomarkers for early OA detection. This transcriptomic analysis reveals key differences between end-stage CTA and primary glenohumeral OA. CTA shows heightened metabolic/protein synthesis activity with less immune-driven inflammation. Under MetS, mitochondrial dysfunction (including *GATD3A* downregulation) and altered Wnt/β-catenin signalling intensify cartilage and bone damage. In contrast, primary OA features strong complement activation, inflammatory gene expression, and collagen remodelling. MetS worsens both conditions via oxidative stress, advanced glycation end products, and ECM disruption—particularly, increased CS/DS degradation. These distinctions support targeted treatments, from antioxidants and Wnt modulators to aggrecanase inhibitors or clusterin augmentation. Addressing specific molecular disruptions, especially those amplified by MetS, may preserve shoulder function, delay surgical intervention, and improve long-term patient outcomes.

## 1. Introduction

Osteoarthritis is a leading cause of disability worldwide, affecting over 527 million people. Despite the socioeconomic burden of OA [1], current conservative treatment is largely limited to pain management and intra-articular glucocorticoid injections [2], underscoring the need for deeper mechanistic insights to guide therapeutic development. Once considered a primarily age-related degenerative condition, OA is increasingly prevalent in younger populations due to factors such as obesity, occupational strain, sports participation, genetic predisposition, and anatomical variations [3], with metabolic disorders contributing to its onset and progression [4,5]. Obesity has traditionally been considered a risk factor due to increased joint loading [6]; however, given the association with hand OA, despite its non-weight-bearing nature, systemic metabolic influences on joint pathology are suspected [7].

MetS is characterized by visceral adiposity, hypertension, dyslipidaemia, and insulin resistance, all of which contribute to a state of chronic low-grade inflammation that disrupts joint homeostasis and accelerates cartilage degradation [4,8,9,10,11,12]. Further, OA patients have a 5.26-fold increased risk of coexisting metabolic syndrome (MetS), independent of obesity [13]. The role of inflammatory gene modulation in OA pathogenesis is well documented [14,15,16], with transcriptomic studies revealing disease-specific genetic signatures in knee and hip OA [17]. However, the molecular landscape of shoulder OA remains largely unexplored.

Emerging transcriptomic analyses suggest that increased cartilage catabolism rather than reduced anabolism, drives MetS-associated OA [18]. For instance, Casagrande et al. identified increased expression of connexin 43 (Cx43), ADAMTS5, and pro-inflammatory cytokines in osteoarthritic cartilage [19], while Aleem et al. reported upregulation of inflammatory mediators such as *CCL3*, *CHST11*, *GPR22*, *PRKAR2B*, and *PTGS2* in shoulder OA compared to instability cases [20].

To further investigate the role of MetS and systemic inflammation in shoulder OA, this study compares subchondral bone and capsular biopsies from patients with primary shoulder OA and cuff-tear arthropathy (CTA), with and without MetS. We hypothesize that CTA will exhibit less pronounced inflammatory gene expression than primary OA, particularly in patients without concomitant MetS.

## 2. Main Points

### 2.1. Inflammation and Mechanical Stress in Joint Degeneration

CTA demonstrates heightened metabolic and protein synthesis activity, reflecting ongoing remodelling, and relatively lower immune-driven inflammation, likely due to altered biomechanics from chronic rotator cuff deficiency. In contrast, primary OA features persistent complement activation, inflammatory gene upregulation, and collagen biosynthetic changes. Both conditions demonstrate distinct transcriptomic patterns under MetS.

### 2.2. MetS’s Impact on OA and CTA

MetS accelerates degeneration via oxidative stress, glycation end products, and dysregulated gene expression. In OA, MetS intensifies collagen crosslinking and chondroitin sulphate degradation, whereas CTA is marked by mitochondrial dysfunction (including *GATD3A* downregulation) and altered Wnt/β-catenin signalling. These differences highlight distinct pathways driving tissue deterioration.

### 2.3. Targeted Therapeutic Opportunities

Addressing inflammation, mitochondrial dysfunction, and ECM breakdown may mitigate disease progression. Antioxidant strategies (e.g., mitochondria-targeted agents) could reduce oxidative damage in CTA. In OA, inhibiting Wnt/β-catenin or aggrecanase, plus using anabolic factors (e.g., sprifermin), may help restore cartilage. Tailoring interventions to each condition’s transcriptomic profile under MetS could preserve shoulder function and delay surgical intervention.

## 3. Results

**Figure 1:**
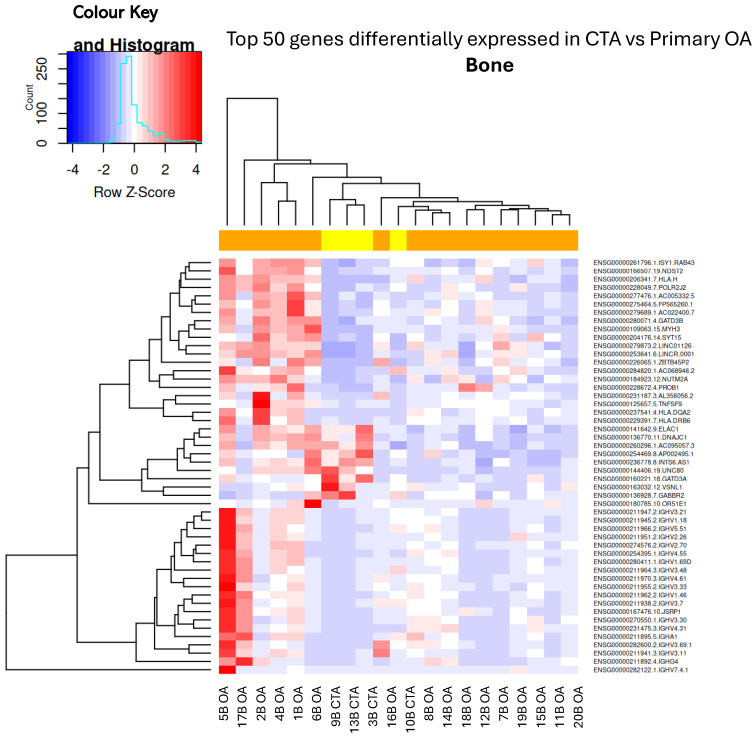
Hierarchical unsupervised clustering (not ordered prior to analysis; genes and samples are positioned based on their observed similarity) gene expression heatmap and colour histogram. Top 50 DEGs in CTA compared with shoulder OA. Bone biopsies for patients 1 through to 20. B = bone, OA = OA, CTA = cuff-tear arthropathy.

The transcriptomic drivers of CTA compared to primary shoulder OA demonstrate a predominance of catabolic and inflammatory genes in subchondral bone biopsies (Table 1 and Figure 1). *GATD3A*, a top differentially expressed gene (DEG), was downregulated in both bone and capsular tissue of study patients with CTA as compared to patients with primary OA (Table 1 and Table 2).

**Figure 2:**
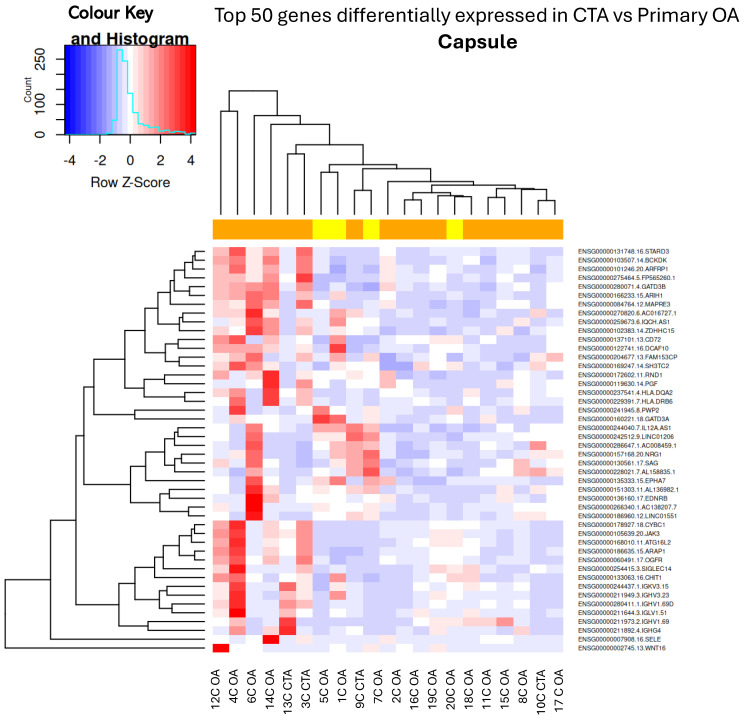
Hierarchical unsupervised clustering (not ordered prior to analysis; genes and samples are positioned based on their observed similarity) gene expression heatmap and colour histogram. Top 50 DEGs in CTA compared with shoulder OA. Capsular tissue biopsies for patients 1 through to 20. C = capsule, OA = OA, CTA = cuff-tear arthropathy.

When comparing capsular tissue biopsies between CTA and primary OA, the downregulation of anabolic genes such as *MAS1*, *ACSM3*, and *LINC01229* was noted, as well as the downregulation of several anti-inflammatory genes (Table 2 and Figure 2).

**Figure 3:**
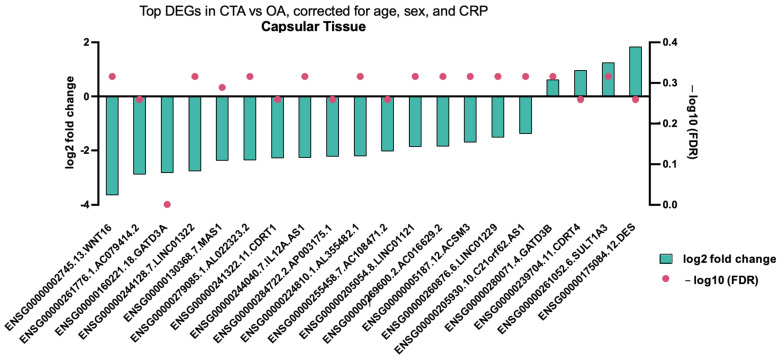
Top DEGs in CTA compared with shoulder OA, adjusted for age, sex, and CRP in capsular tissue biopsies. A single differentially expressed gene (adjusted *p* value < 0.05) is shown.

Comparison of capsular tissue biopsies between CTA and primary OA demonstrated a singular differentially expressed gene, specifically the downregulation of *GATD3A* (Figure 3).

**Figure 4:**
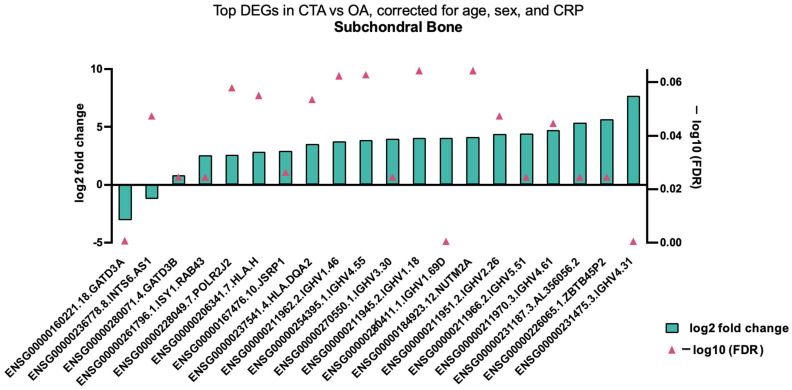
Top DEGs in CTA compared with shoulder OA, adjusted for age, sex, and CRP in bone tissue biopsies.

When analysing specific gene variation in subchondral bone tissue biopsies between CTA and primary OA, several significantly DEGs were demonstrated, including inflammatory genes and those involved in mitochondrial homeostasis, including *GATD3A* (Figure 4). When analysing all tissue types, subchondral bone, capsule, and synovium, a predominance of downregulated anabolic-associated genes was demonstrated, with up- and downregulated inflammatory and catabolic genes also shown (Table 3).

**Figure 5:**
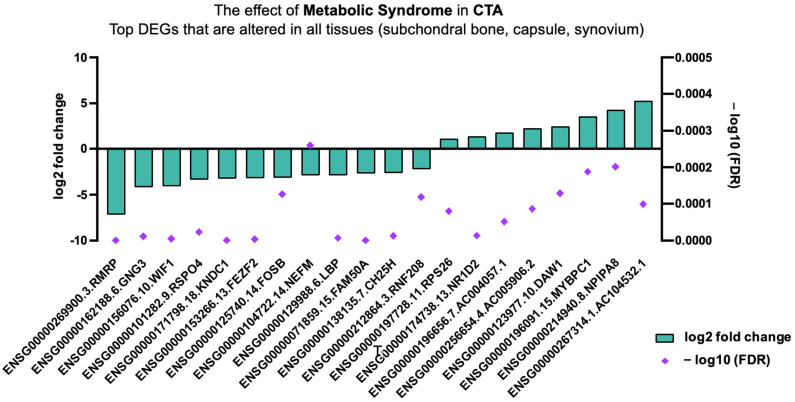
Top DEGs in CTA altered in all tissue types given MetS.

Top DEGs involved in all tissue types biopsied, including subchondral bone, capsule, and synovium in CTA in patients with MetS, are shown in Figure 5. Significantly, DEGs impacted by MetS involve cellular and chondrocyte proliferation, bone mineralization, modulation of WNT/β-catenin signalling, low-grade inflammation, weight gain, and increased cholesterol uptake; see Table 3 and Figure 5.

When analysing the effect of MetS on primary shoulder OA, subchondral bone biopsies analysed with mitch demonstrated upregulation of collagen-associated anabolic pathways (Figure 5).

Subchondral bone biopsies in MetS-associated CTA show differential expression profiles dominated by downregulation of anabolic genes as well as several inflammatory genes, including *ZC3H13*, *DNER*, and *MX1* (Table 4).

Subchondral bone biopsies in MetS-associated primary shoulder OA show a more inflammatory profile; upregulated anabolic and catabolic processes also demonstrated this (Table 5).

**Figure 6:**
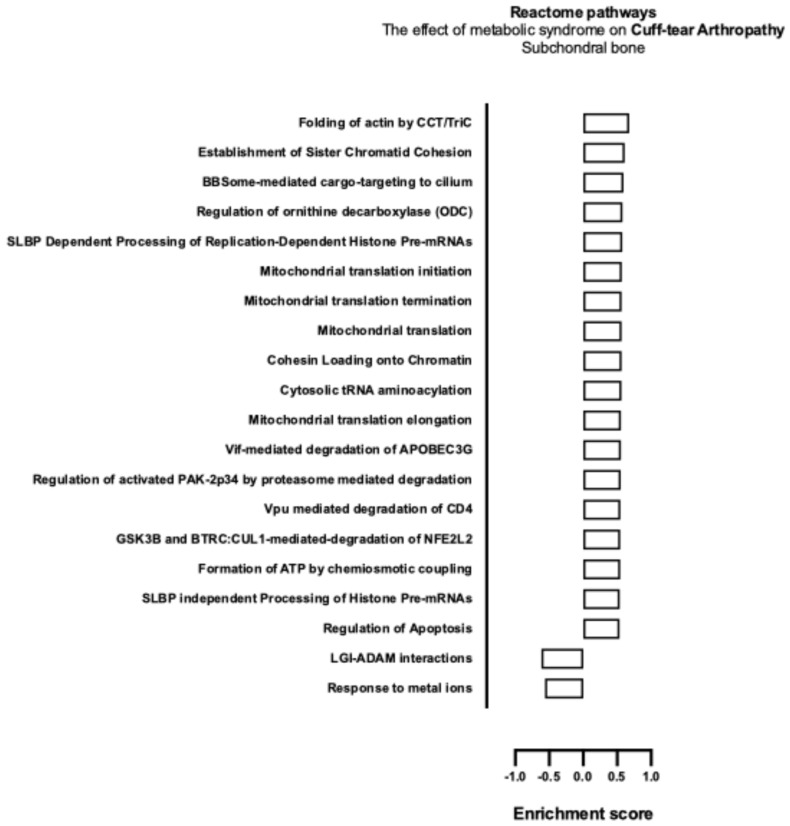
Biological enrichment processes in CTA demonstrating the effect of MetS in subchondral bone biopsies.

**Figure 7:**
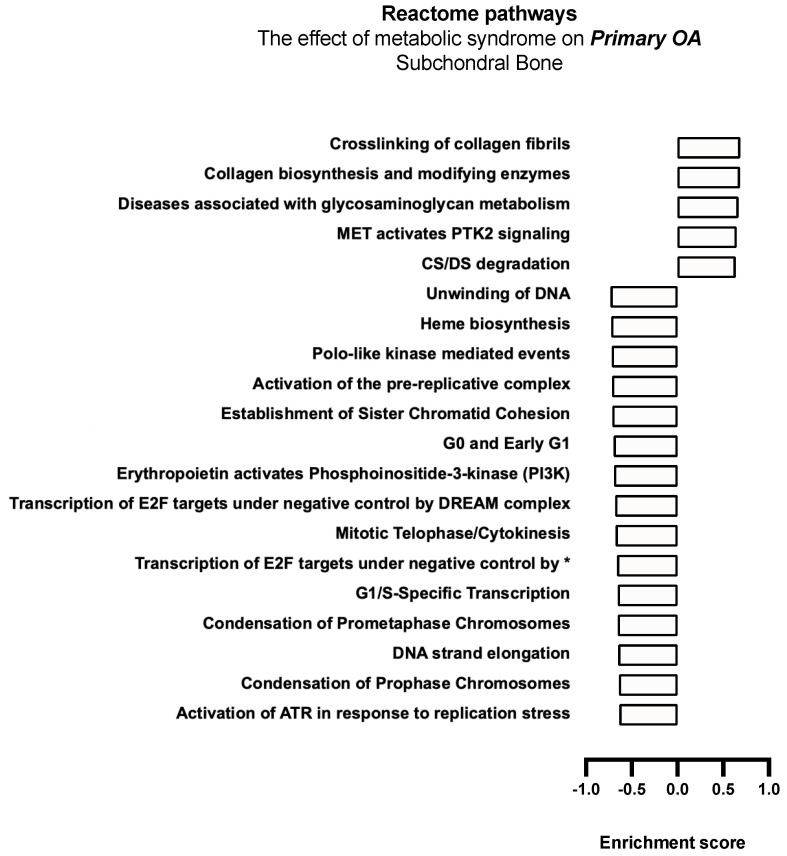
Biological enrichment processes in primary OA demonstrating the effect of MetS in subchondral bone biopsies. * Transcription of E2F targets under negative control by p107 (RBL1) and p130 (RBL2) in complex with HDAC1.

The effect of MetS on CTA and primary shoulder OA with respect to Reactome gene pathway activity as analysed with mitch demonstrated the upregulation of apoptotic pathways (Table 6), those pertaining to energy production, and the maintenance of genomic stability (see Figure 6 and Figure 7).

MetS-associated primary OA demonstrated a significant downregulation of anabolic and cell cycle progression, DNA replication, and DNA repair pathways, including decreased G0 and early G1 Reactome, G1/S transcription, prometaphase chromosomal condensation, DNA unwinding, as well as the transcription of E2F targets under negative control by the DREAM complex pathway, indicating a significant reduction in anabolic processes in subchondral bone biopsies. Upregulation of the chondroitin sulphate (CS) and dermatan sulphate (DS) degradation pathway was also noted (Figure 7).

**Figure 8:**
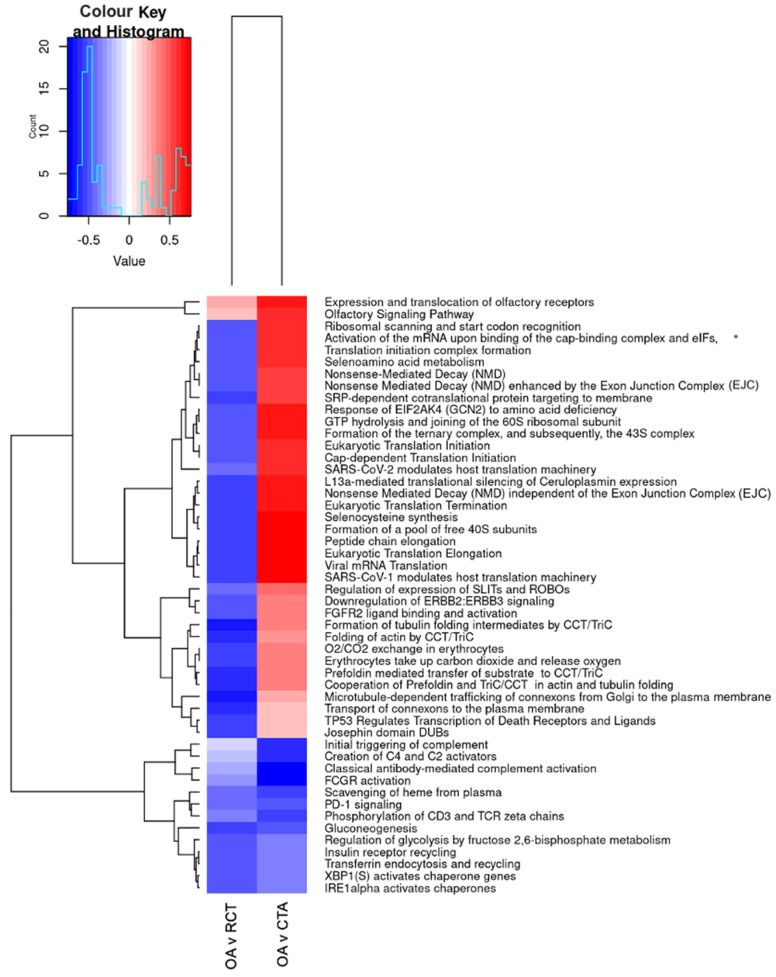
Differential pathway regulation in primary shoulder OA, RCT, and cuff-tear arthropathy (CTA). * Activation of the mRNA upon binding of the cap-binding complex and eIFs, and subsequent binding to 43S. The heatmap displays significantly enriched pathways across tissue comparisons: OA vs. RC (first column, OA v RCT) and CTA vs. OA (second column, OA v CTA). The colour scale represents the extent of pathway upregulation (red) or downregulation (blue), with the histogram in the top left corner showing the distribution of values.

When comparing primary OA to RCT (see Figure 8), OA exhibited upregulation of pathways related to translation and protein synthesis (e.g., ribosomal scanning, translation initiation, and elongation) and RNA processing (e.g., nonsense-mediated decay), while immune activation, complement signalling, glycolysis, and chaperone activity were downregulated.

When comparing CTA to primary OA, similar increases in translation and metabolic activity were observed, with additional upregulation of Wnt/β-catenin signalling and extracellular matrix remodelling pathways (SLIT/ROBO regulation). In contrast, immune response pathways, complement activation, and glucose metabolism were significantly downregulated. These results suggest that CTA exhibits increased metabolic and translational activity, but a reduced immune response compared to primary OA, whereas OA shows greater immune activation and metabolic dysregulation compared to RCTs.

## 4. Discussion

Primary glenohumeral OA involves progressive cartilage wear with typical features like osteophyte formation and subchondral sclerosis, whereas CTA arises specifically following a chronic massive RCT that alters joint biomechanics. Additionally, the loss of the normal sealed (“water-tight”) joint compartment in CTA can compromise synovial nutrition to the cartilage, further accelerating degeneration. In CTA, the deficient rotator cuff causes abnormal glenohumeral loading (e.g., superior migration of the humeral head) and instability, resulting in characteristic wear patterns, as explained by Hamada [95]. Gene regulation in shoulder OA and CTA associated with MetS remains unexplored. This study focuses on the transcriptomic characteristics of these conditions and the role of MetS, revealing that MetS impacts CTA and primary OA differently. In CTA, cartilage anabolism is coupled with upregulated degradation pathways, impaired DNA repair, and disrupted cell cycle progression. In contrast, primary OA involves the upregulation of inflammatory and catabolic genes linked to collagen biosynthesis. Further, MetS in the primary OA group is associated with the upregulation of the chondroitin sulphate (CS) and dermatan sulphate (DS) degradation pathway, responsible for the breakdown of glycosaminoglycans (GAGs), a crucial element of cartilage.

### 4.1. Common Molecular Pathways Involved in Primary OA and CTA

#### ERK1/2 Pathway Activation

The ERK1/2 pathway is activated in both CTA and primary OA, particularly in the presence of MetS. The PI3K/Akt signalling pathway plays a pivotal role in regulating chondrocyte proliferation and apoptosis in OA. The downregulation of the PI3K pathway in MetS-associated OA is both a notable and anticipated finding. Previous studies have proposed that inflammation may inhibit chondrocyte proliferation and the autophagy rate [96]. Ghrelin, a neuropeptide known for its anti-inflammatory properties, has been found to exist in lower levels in individuals with a higher prevalence of MetS [97]. Additionally, ghrelin has been shown to protect against OA by downregulating inflammatory responses [98]. Consequently, it is plausible that mitigating inflammatory mediators associated with MetS could activate the PI3K/Akt pathway, thereby reducing chondrocyte apoptosis.

Further, Simonaro et al. have posited that inflammation significantly contributes to chondrocyte apoptosis through the accumulation of glycosaminoglycans (GAGs) in various bone and joint diseases [99]. Although the precise mechanisms underlying chondrocyte apoptosis in OA remain elusive, in vitro studies indicate that normal chondrocytes exposed to accumulated GAGs exhibit increased levels of the pro-apoptotic lipid ceramide, suggesting a direct impact of GAG fragments on chondrocyte viability [100].

GAGs, including hyaluronan, have recently been implicated in several pathological processes, including the inflammatory response, diet-induced insulin resistance, adipogenesis, and autoimmunity in type 1 diabetes [101]. The observed association between dysregulated GAG metabolism and MetS in patients with primary shoulder OA suggests that the inflammatory effects of MetS contribute to the progression towards end-stage disease, although elucidating the potential causative mechanisms warrants further research.

### 4.2. Distinct Gene Expression and Molecular Pathways Differentiating Primary OA and CTA

CTA is marked by mitochondrial dysfunction and *GATD3A* downregulation. Mitochondrial dysfunction in CTA contributes to oxidative stress, ATP depletion, and cartilage degeneration, making it a promising target for therapeutic intervention. In this condition, inflammatory and catabolic genes are activated alongside mitochondrial gene dysfunction, similarly seen in knee OA [21]. GATD3A plays a role in mitochondrial homeostasis and regulates advanced glycation end products (AGEs), which accumulate when *GATD3A* is knocked down, leading to non-enzymatic glycation of collagen [24,102]. This impairs osteoblast differentiation and bone remodelling [103]. Interestingly, *GATD3A* was not differentially expressed in MetS samples where glycaemic control was poorer, suggesting alternative pathogenic mechanisms in CTA other than non-enzymatic glycation. Human OA chondrocytes with impaired mitophagy exhibit increased ATP production and mitochondrial dysfunction, activating inflammasomes and releasing pro-inflammatory cytokines [22,23], leading to extracellular matrix breakdown [104]. *GATD3A* downregulation intensifies mitochondrial dysfunction, possibly accelerating OA progression by inducing chondrocyte death and activating apoptotic pathways [24,63]. These findings indicate that *GATD3A* downregulation and mitochondrial dysfunction contribute to end-stage CTA.

Enhancing antioxidant defences (e.g., *SOD2* upregulation) and activating SIRT3/AMPK signalling can improve mitochondrial quality control and energy production, protecting chondrocytes from apoptosis [105]. Mitochondria-targeted antioxidants, including Mito-TEMPO, melatonin, quercetin, and dihydromyricetin (DHM), show chondroprotective effects by scavenging ROS and preserving mitochondrial function. DHM specifically enhances antioxidant capacity and matrix synthesis (aggrecan, collagen II) via SIRT3 activation, while resveratrol stabilizes membrane potential and ATP levels, preventing chondrocyte apoptosis [105].

Although not yet standard therapy for OA or CTA, these approaches could mitigate oxidative stress in cuff-tear arthropathy, improve cartilage resilience, and slow disease progression, complementing current surgical and symptom-based treatments.

One of the most notable findings was the increased translation and protein synthesis activity in CTA compared to OA. The upregulation of pathways related to translation initiation, elongation, and ribosomal function suggests heightened chondrocyte or fibroblast activity, likely contributing to greater extracellular matrix (ECM) remodelling in CTA. This may reflect a more dynamic tissue repair or remodelling process, distinguishing it from achondrocytic ECM synthesis and cartilage matrix degradation driven by inflammation in primary OA [106]. Another key difference was the significant downregulation of immune and complement activation pathways (Creation of C4 and C2 activators, classical antibody-mediated complement activation) in CTA. In patients undergoing hip, knee, and shoulder arthroplasty for primary and post-traumatic OA and rheumatoid arthritis, complement factors were detected in osteochondral tissue and were further upregulated in response to IL-1β stimulation, implicating the alternative complement pathway in OA progression [107]. The downregulation of complement system components (C4/C2 activation, classical antibody-mediated complement activation) and Fc receptor (FCGR) signalling in our study suggests that CTA exhibits a lower inflammatory immune response compared to OA. This contrasts with the chronic low-grade inflammation characteristic of OA. Findings from our previous study demonstrated that complement activation is both a localized process and a distinct feature of primary shoulder OA [108]. These findings underscore the distinct role of complement-driven inflammation in OA pathogenesis, setting it apart from CTA, which appears to be driven more by mechanical and metabolic factors rather than immune-mediated inflammation

### 4.3. The Effect of MetS on CTA

#### WIF1 and RSPO4 Downregulation

The Wnt/β-catenin pathway, a crucial regulator of skeletal development and tissue homeostasis, becomes dysregulated in OA. In healthy adult joints, Wnt signalling is relatively quiescent, maintaining a balance between cartilage anabolic and catabolic activities. In OA (including shoulder OA and, as we demonstrate, CTA), excessive Wnt/β-catenin activity has been observed in articular cartilage, subchondral bone, and synovium [109]. Aberrant activation of canonical Wnt signalling drives chondrocytes towards a hypertrophic and catabolic phenotype. Specifically, Wnt target genes such as *WISP-1* (Wnt1-inducible signalling protein-1) are upregulated in human and experimental OA, and they stimulate the production of matrix-degrading enzymes, breaking down collagen and aggrecan [109]. Specifically, Wnt5a (a non-canonical Wnt) has been shown to reduce aggrecan (*ACAN*) expression while increasing MMP-1 and MMP-13, underscoring how Wnt pathways tilt the balance towards matrix catabolism [110].

Alongside enzymatic degradation, excessive Wnt/β-catenin signalling promotes chondrocyte term hypertrophy, which promotes shedding and calcification [111].

Wnt agonists initiate excessive bone remodelling, whilst Wnt antagonists have a protective effect on cartilage [112]. Interestingly, in cuff-tear arthropathy, radiographic analyses note a relative lack of osteophytes and, instead, the presence of subchondral osteopenia and even humeral head collapse in late stages [113]. This suggests that the bone response in CTA may differ from typical OA, potentially due to disuse (from reduced joint loading) or altered mechanotransduction. However, the principle remains that Wnt signalling drives aberrant remodelling: when overactive, it encourages bone formation at joint margins (osteophytes) and when deficient, bone loss can occur. Animal models support that a need for balanced Wnt signalling both excessive activation and complete inhibition of β-catenin in chondrocytes can worsen cartilage outcomes [109]. Thus, too much Wnt causes breakdown, but too little can impair repair and lead to degeneration, indicating a U-shaped relationship. This nuanced understanding is important for therapeutics, as simply blocking Wnt might have unintended consequences if not carefully controlled.

MetS-associated CTA disrupts key pathways involved in cartilage and bone health. *WIF1* and *RSPO4* were downregulated across all tissues, impairing WNT/β-catenin signalling, which is essential for regulating chondrocyte proliferation, bone mineralization, and inflammation. This pathway, crucial in OA pathogenesis [114], remains underexplored in CTA. Additionally, the downregulation of *INTS6.AS1*, *C21orf62.AS1*, and WNT16, a β-catenin inhibitor, further influences WNT signalling in OA [38]. In contrast, the upregulation of *NOTUM*, a WNT inhibitor, in MetS-associated primary OA suggests it may play a protective role in OA [115].

In the early stages of OA, articular chondrocytes exhibit reduced metabolic activity, accompanied by hypertrophy and apoptosis. This is marked by a gradual decline in cartilage-specific gene expression, including *Col2a1* and *aggrecan*, alongside an upregulation of hypertrophic markers like *Runx2* and *Col10a1*. Additionally, there is an increase in the expression of catabolic enzymes such as *Mmp13*, *Adamts4*, and *Adamts5*, which contribute to cartilage breakdown [109]. We found that in CTA, *COL2A1* expression is upregulated within the assembly of collagen fibrils and other multimeric structures pathway, suggesting a potential compensatory response to cartilage degradation. Additionally, genes associated with OA progression, including the hypertrophic marker *COL10A1* and catabolic enzymes *MMP13*, *MMP9*, and *MMP3*, are also upregulated, indicating an active extracellular matrix remodelling process consistent with previous findings in OA pathology [116,117].

### 4.4. Fine-Tuning Wnt/β-Catenin Signaling: Balancing Cartilage Remodeling in OA and CTA

Given Wnt/β-catenin’s central role in joint tissue remodelling, it represents an attractive but challenging therapeutic target. The goal is to dial down the pathological Wnt signalling seen in OA/CTA without abolishing its normal protective effects. Early experimental therapies include small-molecule Wnt pathway inhibitors and biologics that sequester Wnt ligands. One notable example is SM04690 (lorecivivint), an intra-articular Wnt pathway inhibitor previously investigated in knee OA. In preclinical models, SM04690 promoted chondrocyte differentiation and was chondroprotective, suggesting a disease-modifying potential [118]. Phase 2 studies in humans indicated some benefit in pain scores and radiographic stabilization of the joint [119]. Further, using intra-articular Wnt antagonists such as sclerostin (Wnt signalling inhibitor) was shown to have anti-catabolic effects on cartilage in animal models [109]. Whilst no Wnt-targeted therapy has been investigated in shoulder arthritis, these efforts suggest that by fine-tuning Wnt/β-catenin activity, we may be able to curtail cartilage breakdown as well as abnormal bony remodelling in degenerative shoulder conditions. Such therapy would need to be carefully calibrated, given the pathway’s dual-edged nature.

Proteasome degradation of β-catenin through activation of the canonical WNT pathway saturates the destruction complex, allowing β-catenin accumulation and factor-dependent transcription [120]. Future studies should seek to delineate the biomolecular mechanism of transcription-related outcomes on cartilage homeostasis. In contrast to *GATD3A*-related mitochondrial dysfunction in end-stage CTA, WNT/β-catenin signalling acts in primary OA progression due to the disruption of the balance of its regulatory factors. A precise balance of WNT signalling is crucial for cartilage homeostasis, as both suppression and excessive β-catenin activation contribute to cartilage degradation in OA [110]. *WISP1*, a key regulator of Wnt signalling, is elevated in OA and promotes cartilage breakdown by inducing *MMP-3*, *MMP-13*, *ADAMTS-4*, and *ADAMTS-5* [110].

### 4.5. Impaired Metabolic and Cellular Stress Responses

The intricate interplay of differentially expressed pathways in CTA in patients with MetS reveals chronic inflammation, metabolic dysregulation, and oxidative stress: key features of both conditions. In MetS, oxidative stress and metabolic overload impair mitochondrial function and protein folding [121], reducing ATP production and contributing to cellular dysfunction and disease progression. This oxidative stress also compromises mitochondrial function in OA, reducing energy levels and increasing apoptosis in chondrocytes, which accelerates cartilage degradation [122].

In CTA, we demonstrated the upregulation of ornithine decarboxylase (ODC) and the downregulation of cellular response to metal ions, highlighting active cellular stress responses in this condition. Hypoxia-associated pathways, positively enriched in CTA, suggest an adaptive response to low oxygen levels, as has been demonstrated in early rotator cuff disease [123]. Key pathways differentially expressed in CTA, including mitochondrial translation, ATP formation, the degradation of NFE2L2 (Nrf2) by GSK3B and BTRC, and apoptosis regulation, are all influenced by hypoxia and oxidative phosphorylation [124,125,126,127]. The stress response protein Nrf2, known for its antioxidative and anti-apoptotic roles in osteoarthritic chondrocytes, is considered a potential therapeutic target for managing OA [126,128]. Further, disrupted DNA replication and repair processes lead to genomic instability and impaired cell proliferation [129], which we have demonstrated to be features of CTA, given MetS with the differential expression of the establishment of sister chromatid cohesion, cohesin loading onto chromatin, and SLBP-dependent processing of replication-dependent histone pre-mRNAs.

### 4.6. Hypoxia-Responsive Pathways and Emerging Pharmacological Options for Shoulder OA

The activation of those molecular pathways responsive to hypoxic conditions may signal cellular stress and initiate physiological adaptations aimed at coping with reduced oxygen availability. However, the dysregulation or overactivation of these pathways may create a detrimental feedback loop, perpetuating or exacerbating hypoxia. In the near term, several pharmacologic agents being trialled for OA in general could be applied to shoulder joints. Antioxidant and mitochondria-targeted drugs could reduce oxidative cartilage damage, as evidenced by compounds like melatonin and taurine improving chondrocyte survival and matrix synthesis in preclinical models [105].

### 4.7. The Effect of MetS on Primary OA

#### MetS Upregulates *ACAN*, *PANX3*, *CLU*, and *VAT1L*

Aggrecan is the major proteoglycan of articular cartilage, responsible for the tissue’s compressive stiffness and hydration. In shoulder OA, as in other forms of OA, the aggrecan content is progressively lost from the cartilage matrix due to enzymatic degradation [130]. Aggrecanases (primarily ADAMTS-5 and ADAMTS-4) cleave aggrecan, leading to depletion of this key molecule and loss of cartilage resilience [130]. Wnt/β-catenin overactivation exacerbates this problem by upregulating those aggrecan-degrading enzymes [109] and by downregulating expression in chondrocytes [110]. The end result is a vicious cycle: cartilage with reduced aggrecan is more vulnerable to mechanical stress, which, in turn, can further injure chondrocytes and perpetuate inflammation.

In MetS-associated OA, several genes are upregulated, reflecting complex interactions between cartilage regulation, inflammation, and metabolism. *ACAN*, known for its anabolic role in cartilage, is promoted by MIA, which inhibits ERK signalling [88]. Although heightened *ACAN* expression is observed in shoulder OA linked to MetS, contrasting with reduced SOX9 activation in knee OA [131], increased *ACAN* expression does not always signify proper cartilage maintenance. It also indicates increased catabolic processes affecting aggrecan [132].

### 4.8. Potential Therapeutic Strategies Targetting Aggrecan and PANX3 in Osteoarthritis

To preserve and restore aggrecan in arthritic cartilage, two strategies are being explored, inhibiting aggrecan breakdown and stimulating aggrecan synthesis. On the anti-catabolic front, pharmaceutical companies have developed ADAMTS-5 inhibitors as potential disease-modifying OA drugs. For example, GLPG1972/S201086 is an orally available ADAMTS-5 inhibitor that was tested in a phase 2 trial (the ROCCELLA study) for knee OA [133]. The rationale is that blocking the major aggrecanase will slow aggrecan depletion and cartilage erosion. Although most trials so far have focused on weight-bearing joints, the same principle could apply to the shoulder. Another anti-catabolic approach is using biologic agents (e.g., monoclonal antibodies or nanobodies) against aggrecanases. Specifically, M6495, a nanobody targeting ADAMTS-5, has shown safety in early trials [134]. Further, growth factor therapies endeavour to enhance chondrocyte production of aggrecan and collagen. Sprifermin (rhFGF18) is an anabolic therapy that has reached clinical trials, and stimulates chondrocyte proliferation and matrix synthesis, increasing cartilage thickness in a dose-dependent manner while also reducing catabolic enzyme activity in knee OA [135]. By promoting aggrecan and collagen II deposition, sprifermin effectively attempts to tilt the scales towards joint repair. Though these interventions are not routinely available, they demonstrate the translational potential of research aimed at finding effective disease-modifying OA drugs (DMOADs). Further exploration targeting aggrecanase inhibition represents a promising avenue for disease-modifying treatments in OA [133].

Pannexin 3 is a channel-forming glycoprotein expressed in cartilage and bone cells, involved in mechanotransduction and ATP/calcium signalling between cells. Recent studies have identified PANX3 as a pro-catabolic factor in OA pathology. Notably, *PANX3* expression is low in healthy articular cartilage but is strongly upregulated in osteoarthritic cartilage lesions in both mice and humans [91]. Specifically, mice lacking *PANX3* in cartilage were resistant to developing post-traumatic OA after injury (destabilization of the meniscus) [91]. This suggests that PANX3 contributes to cartilage degeneration under stress conditions, possibly by facilitating the release of inflammatory ATP signals or by altering chondrocyte mechanosensitivity. Interestingly, the role of PANX3 may be context-dependent, with some evidence indicating that while PANX3 drives injury-induced OA, its absence in natural ageing may have complex effects, as *PANX3* knockout mice showed accelerated spontaneous cartilage degeneration [136]. Nonetheless, in the setting of relative joint concentricity, such as MetS-OA in our study, *PANX3* upregulation, we posit, promotes the joint’s degenerative response. PANX3 is vital for chondrogenesis and osteogenesis [91], activating PI3K/AKT signalling and stimulating connexin 43 (Cx43), a key pro-inflammatory factor, particularly in shoulder OA [19,90,137,138].

### 4.9. Novel Targets and Therapeutic Approaches: Inhibiting PANX3/Cx43 and Harnessing Clusterin

Targeting Panx3 and Cx43 might offer therapeutic options to mitigate inflammation in primary shoulder OA. The success in preventing OA progression without altering skeletal structure in *Panx3* knockdown mice highlights its potential for gene therapy [91]. PANX3 represents a novel target for chondroprotective therapy, wherein inhibiting this channel could slow cartilage breakdown. Pharmacologically, drugs that block pannexin channels are being considered. Probenecid is known to broadly inhibit pannexin 1 channels and has been used in research to inhibit pannexin-mediated signalling [139]. The future development of selective PANX3 inhibitors or blocking antibodies might provide a more targeted approach. From a genetic standpoint, experimental methods like siRNA or gene editing could knock down PANX3 expression in joint tissues. The proof-of-concept stems from the Panx3 knockout mice previously discussed, which showed cartilage protection in injury models [91]. The translational challenge will be to achieve tissue-specific inhibition in the human joint, for example, an intra-articular injection of a PANX3 antisense oligonucleotide or a viral vector carrying a dominant-negative PANX3. As these are early-stage ideas, safety and off-target effects (given PANX3’s roles in normal physiology, including bone growth) must be carefully evaluated.

Clusterin (CLU), a chaperone protein, is associated with joint inflammation and synovitis, particularly in obesity [49,140]. It plays a protective role in early OA but decreases in advanced stages [141], acting as a molecular chaperone and cellular stress protein facilitating the clearance of cellular debris, inhibiting protein aggregation, and modulating apoptosis and inflammation [142]. Under inflammatory stress (e.g., exposure to IL-1β), chondrocytes increase the production of clusterin, suggesting a stress response; however, evidence suggests that in late-stage OA cartilage, overall clusterin expression can be reduced through chondrocyte senescence or the exhaustion of this protective mechanism [141].

The functional importance of CLU in cartilage was highlighted by a recent in vitro study: adding exogenous clusterin to human OA chondrocytes significantly blunted inflammation and cell death. Clusterin treatment suppressed the production of inflammatory mediators (NO, IL-6, TNF-α) and the pro-apoptotic effector caspase-3, while upregulating key anabolic genes like *SOX9* and *ACAN* aggrecan [143]. It also downregulated catabolic and inflammatory genes (NF-κB, MMP-13, IL-6) via activation of the pro-survival PI3K/Akt pathway [143].

These findings illustrate that clusterin is a natural dampener of OA pathogenesis, counteracting the cartilage-destructive milieu. As OA progresses, chondrocytes shift from a stable phenotype (*COL2A1*, *ACAN*) to a hypertrophic state (*COL10A1*, *MMP13*) liable to shedding [144,145].

### 4.10. CLU as a Potential Synovial and Systemic Biomarker: Therapeutic Pathways in MetS-Associated OA

CLU silencing in OA chondrocytes has been shown to increase *MMP13* and *COL10A1* expression, suggesting a protective role in maintaining chondrocyte stability [146]. Further, anti-inflammatory properties position CLU as a potential synovial and systemic biomarker of OA [147]. Augmenting clusterin levels or activity in the joint is a promising therapeutic concept to harness its chondroprotective effects. Pharmacologically, one might envision administering recombinant clusterin protein into the joint space to supplement what osteoarthritic chondrocytes may lack. Given that CLU is secreted and works extracellularly (and at the cell surface), an injected form might directly influence the cartilage surface and synovium. Another approach may be to use small molecules to upregulate the *CLU* gene expression in chondrocytes. From a genetic therapy perspective, gene delivery of *CLU* via viral vectors could ensure a sustained local increase in clusterin. For instance, an adeno-associated virus (AAV) carrying the human *CLU* gene could be injected into the shoulder joint, leading to chondrocytes and synoviocytes secreting higher amounts of clusterin protein. Such gene therapy approaches are speculative but increasingly feasible as techniques improve; notably, gene therapies for joint diseases (like the IL-1 receptor antagonist gene for arthritis) have been tested in clinical trials. Encouragingly, the recent in vitro data make a strong case that boosting clusterin could protect cartilage, with the authors concluding that *CLU* is a promising target for therapeutic intervention in OA [143]. Moving this forward will require in vivo studies to see if clusterin delivery can indeed slow or reverse cartilage degeneration in animal models of shoulder arthritis. If successful, clusterin-based therapy might provide an anti-inflammatory and anti-apoptotic treatment option distinct from traditional steroids or NSAIDs.

Adiponectin, which is typically beneficial in MetS, is paradoxically linked to increased OA risk [148,149]. *VAT1L*, associated with bone metabolism, is upregulated in MetS-associated OA. Its relationship with single-nucleotide polymorphisms, which correlate with higher plasma adiponectin levels, may reveal its role in cartilage regeneration and potential as a biomarker for OA progression [76]. These findings highlight the intricate balance between anabolic and catabolic processes in MetS-associated OA, suggesting that targeting specific genes involved in cartilage regulation, inflammation, and metabolism could offer new therapeutic approaches for managing disease progression and severity.

### 4.11. Pathways in MetS and Primary Shoulder OA

In MetS-associated OA, we observed downregulation of G0, early G1, and G1/S transcriptional activity in subchondral bone, indicating impaired chondrocyte cell cycle progression. This aligns with existing knowledge that WNT/β-catenin signalling regulates chondrocyte proliferation by halting the cell cycle at the G1/S restriction point in a glucosamine-dependent manner [114]. WNT/β-catenin also regulates *Cx43* expression, a gene linked to inflammation in OA [90]. Furthermore, variations in the *FRZB* gene, especially in women with hip OA, reduce WNT pathway antagonism, further disrupting cartilage maintenance [150].

MetS impacts the expression of extracellular matrix (ECM) biosynthetic pathways. Among these, the collagen fibril crosslinking pathway emerges as the most positively enriched pathway affected by MetS in primary OA. This pathway is crucial for organising the ECM, serving as a cornerstone for maintaining the mechanical strength and structural integrity of various tissues throughout the body. Understanding how MetS alters these pathways can lead to novel interventions aimed at restoring ECM homeostasis and potentially mitigating the severity of shoulder OA. Research conducted by Steinberg et al. (2021) on cartilage and synovial biopsies from patients undergoing total knee replacement revealed distinct disease processes that are specific to the tissue type. The analysis indicated that the synovium exhibited unique expression profiles related to ECM receptor interactions and focal cell adhesion pathways [151], aligning with our finding of pronounced upregulation of collagen fibril crosslinking pathways in MetS-OA. This excessive crosslinking leads to the stiffening of cartilage, diminishing its flexibility at the articular surface and the cartilage–bone interface [152]. Such alterations impair the cartilage’s capacity to absorb mechanical stress, resulting in accelerated catabolism of chondrocytes while simultaneously hindering their anabolic processes in OA [153]. This dual effect exacerbates the progression of OA, highlighting the need for targeted therapeutic strategies that can mitigate these biochemical changes and restore normal cartilage function.

Moreover, the accumulation of advanced glycation end products (AGEs) is a critical factor in this context. AGEs, which can form crosslinks with collagen, contribute to the “age-related failure” of collagen in human articular cartilage, rendering it more susceptible to structural damage [154]. Notably, elevated levels of AGEs have been documented in individuals with MetS, potentially exacerbating joint space narrowing in knee OA [155]. Our observations underscore the significant differential expression of collagen fibril crosslinking pathways in primary shoulder OA associated with MetS, highlighting a potential link between AGEs and the progression of OA.

### 4.12. Chondroitin Sulphate Degradation Pathways in MetS-Associated OA Pathogenesis

The downregulation of critical pathways in MetS-associated OA is expected to adversely affect DNA repair mechanisms, cell cycle progression, and transcriptional regulation, while also disrupting cellular metabolism in end-stage shoulder OA. These alterations contribute to diminished chondrocyte functionality, increased apoptosis, impaired cartilage repair processes, and accelerated joint degeneration. Furthermore, the degradation pathways of chondroitin sulphate/delta-sulphated (CD/DS) were upregulated in MetS-associated OA, influencing various aspects of cartilage homeostasis and joint function. Hyperglycaemia associated with MetS can alter the function of the disaccharide-containing CS and DS and is linked to altered ECM function in a range of tissues that drive complications associated with MetS [156]. Despite the substantial differences between these diseases, our findings indicate that the upregulation of CD/DS degradation pathways due to MetS implies a role for hyperglycaemia and/or inflammatory pathways in this process.

### 4.13. Translational Insight, GLP-1 Agonists

Recent evidence posits the beneficial role GLP-1 Agonists, used in the treatment of diabetes, may have in alleviating OA progression through interactions between cartilage, synovium, and subchondral bone through homeostatic mechanisms via the gut–bone axis, and locally by suppressing pro-degradative and inflammatory enzymatic processes [157].

### 4.14. Limitations

This study is the first transcriptomic analysis comparing gene expression in MetS-associated primary OA and CTA patients as well as 25 patients undergoing RCR as a comparator group; we analysed 45 patients in total and 85 biopsies, one of the largest datasets of its kind. Due to ethical constraints, samples were limited to patients requiring shoulder arthroplasty, reducing variance in confounding factors like age and occupational demands. Future studies should validate findings with larger, more diverse cohorts.

Technical constraints capped RNA sequencing and PCR at 15 million reads per sample, limiting comprehensive sequence coverage. Full diversity requires up to 500 million reads, exceeding industry standards [158]. Bulk RNA-seq measures average gene expression across samples, unlike single-cell RNA-seq, which accounts for cellular heterogeneity. Despite this, RNA-seq is a robust method, often eliminating the need for RT-qPCR validation, as demonstrated by strong concordance in prior studies [159,160].

Our data reflect transcriptomic profiles of end-stage OA and CTA in patients undergoing joint replacement, leaving early disease mechanisms uncertain. Variable gene expression across a lifetime, as seen with clusterin, remains unexplored [141]. Nevertheless, our findings establish a foundation for future research on early OA and chronic RCTs, potentially aiding in early diagnosis and targeted intervention, particularly for MetS-associated pathologies.

## 5. Materials and Methods

Clinical Trial Number: This investigation has been registered with the Australian New Zealand Clinical Trials Registry (ANZCTR), registered on the 26 March 2018, registration number 12618000431224, accessible from https://anzctr.org.au/Trial/Registration/TrialReview.aspx?id=374665&isReview=true.

### 5.1. Patients

This was a prospective, longitudinal, multi-centre clinical and laboratory-based study. A total of 20 patients with shoulder OA undergoing either anatomic or reverse total shoulder replacement were included. Eligible patients had at least six months of symptoms with radiographic evidence of OA. Those diagnosed with cuff-tear arthropathy (CTA) based on the Hamada classification [161] underwent reverse total shoulder replacement (*n* = 4), whereas those without full-thickness rotator cuff tears (RCTs) diagnosed on ultrasound or MRI underwent anatomic total shoulder replacement (*n* = 16). Patients with inflammatory arthritis, prior shoulder fractures or dislocations, or corticosteroid injections within three months were excluded.

Given the well-established progression from chronic RCTs to CTA, a control group of 25 patients with chronic RCTs was included to provide a comparative baseline. These patients had symptomatic, full-thickness RCTs for over six months, confirmed by ultrasound or MRI. Exclusion criteria included inflammatory arthritis, prior shoulder trauma, or corticosteroid injection within three months.

Written informed consent was obtained from all patients. Surgeries were performed by three specialized shoulder surgeons, who obtained tissue biopsies from subchondral bone, capsule, and synovial tissue in the primary OA and CTA groups, aggregating 60 separate tissue samples for analysis. In the RCT group, capsular tissue (3 × 3 mm) was collected to ensure comparable tissue types were analysed across pathologies in bioinformatic analysis.

This study design enables comparative analysis of molecular differences between primary OA, CTA, and chronic RCTs, providing insights into disease progression and distinct pathological mechanisms.

### 5.2. Clinical Data Collection

Data on preoperative patient characteristics, including age, sex, and smoking status, were collected along with clinical data, including body mass index (BMI), American Society of Anesthesiologists (ASA) physical status score [162], presence or absence of MetS and its constituents, and preoperative renal function.

### 5.3. RNA Extraction and Transcriptomics Analysis

All tissues underwent RNA extraction using a commercially available kit (Qiagen, Hilden, Germany), and the quantity and quality of RNA were measured using the Agilent Bioanalyzer (Santa Clara, CA, USA). The Illumina TruSeq RNA preparation kit (San Diego, CA, USA) was used to generate a library from 1 μg of RNA. Briefly, RNA was reverse transcribed to generate complementary DNA (cDNA), with reverse transcriptase supplemented with actinomycin-D during the first strand, followed by second strand synthesis. Double-stranded cDNA libraries were then sequenced using Illumina NovaSeq at the Deakin Genomics Facility, Geelong, Australia.

The resulting fastq files underwent QC analysis using FastQC (v0.12.1) and MultiQC (v1.26) tools [163,164]. Fastq files were quality trimmed using Skewer to remove 3′ ends with quality scores lower than 10 [162]. Trimmed reads were then mapped to the human Gencode transcriptome (version 378) [165] using Kallisto (v0.48.0), a transcript isoform-aware aligner [166]. Transcript counts were read into R (v4.4.1) and aggregated to the gene level for statistical analysis. This analysis was conducted using DESeq (v1.40.2) [167] and corrected for demographic information such as sex, age, and CRP. Principal component analysis was used to identify outliers and potential confounders.

Functional enrichment analysis approaches were used to determine pathway-specific alterations in gene expression, to provide new insights into the pathogenesis of OA and CTA. The pathway database was downloaded from Reactome [168]. The DESeq2 t-statistic was used to score differential expression for each gene, and the enrichment was evaluated using the mitch bioconductor package (v1.16.0) [169].

Differentially expressed genes (DEGs) and pathways (log fold change (LFC) ± 1.5 and FDR ≤ 0.01) were examined and compared in capsular tissue, subchondral bone, and capsule within and between the OA and CTA groups. Differential analysis with DESeq2 was performed, taking into consideration the sex, age, and CRP of the patients. Multi-contrast pathway analysis with mitch was undertaken, referencing Reactome pathways to identify differentially expressed pathways enriched in OA, and CTA in patients with and without MetS [169]. Further pathway analysis with mitch was undertaken, referencing Reactome pathways to identify differentially expressed pathways enriched in capsular tissue, comparing RCR to OA, and CTA. Nonparametric Wilcox tests were undertaken to reduce the batch effect. Pathway analysis was undertaken using results from DESeq2 and Wilcox testing. The code is available from https://github.com/markziemann/shoulder-instability-osteroarthritis/blob/main/metab_syndr.Rmd, accessed on 8 November 2024.

## 6. Conclusions

In conclusion, this study elucidates distinct transcriptomic signatures and molecular pathways differentiating primary glenohumeral OA from CTA, with particular emphasis on the modulatory role of MetS. Our findings highlight significant divergences, notably CTA’s association with mitochondrial dysfunction, impaired DNA repair, increased extracellular matrix remodelling, and reduced immune-mediated inflammatory responses compared to primary OA, which demonstrates prominent inflammatory gene activation and heightened extracellular matrix degradation through collagen biosynthesis pathways.

MetS exerts differential effects in these conditions. In CTA, MetS contributes to the disruption of WNT/β-catenin signalling via the downregulation of *WIF1* and *RSPO4*, impacting cartilage and bone homeostasis. Conversely, in primary OA, MetS upregulates critical factors, including *ACAN*, *PANX3*, *CLU*, and *VAT1L*, enhancing cartilage catabolism, inflammation, and extracellular matrix stiffness, mediated by the accumulation of advanced glycation end products (AGEs). Notably, the chondroitin and dermatan sulphate degradation pathways, essential for cartilage integrity, are significantly upregulated in primary OA with MetS, underscoring the pathogenic role of metabolic dysregulation.

From a translational perspective, our study highlights promising therapeutic targets: mitochondrial antioxidants to combat oxidative stress in CTA; WNT pathway modulation through agents like lorecivivint and sclerostin for balancing cartilage remodelling; aggrecanase inhibitors to prevent cartilage breakdown; and clusterin augmentation for its chondroprotective potential. Additionally, targeting PANX3-mediated inflammatory signalling presents a novel intervention pathway. The nuanced understanding of gene expression and metabolic disruptions identified here provides a foundational platform for future research into early diagnostic markers and personalized, disease-modifying therapies for both primary shoulder OA and CTA, particularly within MetS-associated populations.

## Figures and Tables

**Table 1:** Top DEGs in subchondral bone for patients with CTA compared to primary OA, corrected for age, sex, and CRP. Likely gene effect is described with pathophysiological description and references shown.

Genes	Regulation	Likely Effect of Gene	Pathophysiological Description	Log2FoldChange	−log10 (FDR)	References
IGHV4.31	Upregulated	Inflammatory	Adaptive immune response	7.691303	0.0005410	
IGHV1.69D	Upregulated	Inflammatory	Adaptive immune response	4.073358	0.0005410	
GATD3A	Downregulated	Catabolic Inflammatory	Downregulation → mitochondrial dysfunction - Inflammatory cascade (IL-1β, IL-18, TNF-α) - Chondrocyte apoptosis - Cartilage catabolism (IL-6, MMP-3, MMP-9, MMP-13, ADAMTS5)	−3.070696	0.0006993	[21,22,23,24,25]
ISY1.RAB43	Upregulated	Unclear	Unclear	0.5688546	0.0244115	
IGHV5.51	Upregulated	Inflammatory	Adaptive immune response	4.437549	0.0244115	
ZBTB45P2	Upregulated	Unclear	Unclear	5.661125	0.0244115	
GATD3B	Upregulated	Unclear	Unclear	0.842649	0.0244115	
IGHV3.30	Upregulated	Inflammatory	Adaptive immune response	3.974320	0.0244115	
AL356056.2	Upregulated	Inflammatory	Potentially regulate gene expression and facilitate inflammatory tumour immune microenvironment	5.391960	0.0244115	[26]
JSRP1	Upregulated	Unclear	Unclear	2.933264	0.0263392	
IGHV4.61	Upregulated	Inflammatory	Adaptive immune response	4.750504	0.0445601	
IGHV2.26	Upregulated	Inflammatory	Adaptive immune response	4.409251	0.0473066	
INTS6.AS1	Downregulated	Anti-inflammatory	Reflects INTS6 expression, which promotes WIF1 expression and inhibits WNT signalling in malignancy	−1.213004	0.0473066	[27]
HLA.DQA2	Upregulated	Inflammatory	Autoimmunity	3.544540	0.0535342	
HLA.H	Upregulated	Unclear	Unclear	2.875753	0.0550853	
POLR2J2	Upregulated	Unclear	Unclear	2.618078	0.0579623	
IGHV1.46	Upregulated	Inflammatory	Adaptive immune response	3.745839	0.0623642	
IGHV4.55	Upregulated	Inflammatory	Adaptive immune response	3.875863	0.0628543	
IGHV1.18	Upregulated	Inflammatory	Adaptive immune response	4.064690	0.0643239	
NUTM2A	Upregulated	Catabolic Inflammatory	Decreased level of complimentary antisense RNA NUTM2A-AS1 regulates miR-183-5p/TGFA pathway - Reduced IL-1β-mediated chondrocyte apoptosis and inflammation	4.137140	0.0643239	[28]

**Table 2:** Top DEGs in capsular tissue for patients with CTA compared to primary OA, corrected for age, sex, and CRP.

Genes	Regulation	Likely Effect of Gene	Pathophysiological Description	Log2FoldChange	−log10 (FDR)	References
*GATD3A*	Downregulated	Catabolic Inflammatory	As above	−2.8201856	0.0008253	
*CDRT1*	Downregulated	Unclear	Unclear	−2.2904291	0.2588321	
*AC079414.2*	Downregulated	Unclear	Unclear	−2.8887873	0.2588321	
*DES*	Upregulated	Unclear	Maintains normal mitochondrial function in skeletal muscle	1.8294800	0.2588321	[29]
*AP003175.1*	Downregulated	Unclear	Unclear	−2.2330722	0.2588321	
*CDRT4*	Upregulated	Unclear	Unclear	0.9557273	0.2588321	
*AC108471.2*	Downregulated	Unclear	Unclear	−2.0403979	0.2588321	
*MAS1*	Downregulated	Anabolic	Downstream activation of ERK1/2 signalling - Angiogenesis	−2.3785438	0.2883475	[30]
*AC016629.2*	Downregulated	Unclear	Unclear	−1.8487647	0.3150250	
*ACSM3*	Downregulated	Anabolic	Downregulation in acute myeloid leukemia → increased IGF2BP2 expression - Increased cell proliferation - Decreased cell apoptosis	−1.7109043	0.3150250	[31]
*IL12A.AS1*	Downregulated	Anti-inflammatory	Decreased expression in SLE → T follicular regulatory cell activation	−2.2720054	0.3150250	[32]
*C21orf62.AS1*	Downregulated	Anti-inflammatory	Activation of WNT signalling in malignancy	−1.3878887	0.3150250	[33]
*LINC01121*	Downregulated	Inflammatory	Modulation of MMP-16 expression - Inflammatory cytokine (IL-6, TNF-α, IL-1β) production in intervertebral disc degeneration	−1.8717111	0.3150250	[34]
*AL022323.2*	Downregulated	Unclear	Unclear	−2.3579100	0.3150250	
*LINC01322*	Downregulated	Unclear	Unclear	−2.7691623	0.3150250	
*SULT1A3*	Upregulated	Anabolic	Potential regulation of bone response to thyroid hormone and oestrogen in osteoblast	1.2454267	0.3150250	[35]
*LINC01229*	Downregulated	Anabolic	Downregulation → increased MAF expression - Increased osteoblast differentiation through interaction with Runx2	−1.5291981	0.3150250	[36,37]
*AL355482.1*	Downregulated	Unclear	Unclear	−2.2142403	0.3150250	
*WNT16*	Downregulated	Anti-inflammatory	WNT signalling inhibition in the absence of other WNT ligands Otherwise lubricin-dependent transient WNT signalling activator	−3.6505808	0.3150250	[38]
*GATD3B*	Upregulated	Unclear	Unclear	0.6112931	0.3150250	

**Table 3:** Top DEGs that are altered in all tissues (subchondral bone, capsule, synovium) of MetS-associated CTA.

Genes	Regulation	Likely Effect of Gene	Pathophysiological Description	Log2FoldChange	−log10 (FDR)	References
KNDC1	Downregulated	Anabolic	Downregulation induces ERK 1/2 signalling - Increased cell proliferation - Decreased cellular senescence	−3.274925	0.0010439	[39,40]
FAM50A	Downregulated	Anabolic	Interaction with Runx2 - Ameloblast differentiation - Bone mineralization	−2.711501	0.0010439	[41]
RMRP	Downregulated	Anabolic	Downregulation regulates miR-206/CDK9 axis - Chondrocyte proliferation - Suppression of chondrocyte apoptosis	−7.205598	0.0016968	[42]
FEZF2	Downregulated	Anabolic	Wnt/β-catenin signalling activation - Neuronal differentiation	−3.203489	0.0139739	[43]
WIF1	Downregulated	Anabolic Anti-inflammatory	Wnt/β-catenin signalling suppression → reduced ROS and MMP levels - Chondrocyte proliferation - Suppress chondrocyte apoptosis	−4.115397	0.0165503	[44]
LBP	Downregulated	Inflammatory	Low-grade inflammation - Worsened post-traumatic OA cartilage destruction	−2.881010	0.0182503	[45]
GNG3	Downregulated	Unclear	Knockdown in mice models leads to decreased weight gain and is potentially implicated in the development of diabetes	−4.216226	0.0246764	[46]
CH25H	Downregulated	Catabolic Inflammatory	Increased LOX1-mediated cholesterol uptake and CH25H-CYP7B1-RORα axis regulation - Cartilage degradation	−2.647419	0.0246764	[47]
NR1D2	Upregulated	Anabolic	Suppression of miR-128a expression - Decreased suppression of cartilage anabolic factors - Increased expression of aggrecan, collagen type II, and SOX9	1.394920	0.0246764	[48]
RSPO4	Downregulated	Anabolic Anti-inflammatory	Modulation of WNT/β-catenin signalling - Abnormal bone mineralization	−3.360214	0.0392417	[48,49]
AC004057.1	Upregulated	Others (unclear)	Unclear	1.831635	0.0821972	[50]
RPS26	Upregulated	Catabolic	Modulation of p53 transcriptional activity - Cell cycle arrest	1.162675	0.1167178	
AC005906.2	Upregulated	Unclear	Unclear	2.300732	0.1167178	
AC104532.1	Upregulated	Unclear	Unclear	5.307702	0.1247431	
RNF208	Downregulated	Catabolic	Vimentin degradation - Suppression of triple-negative breast cancer metastasis	−2.221922	0.1336789	[51]
FOSB	Downregulated	Anabolic	ERK1/2 signalling activation - Osteogenic differentiation of mesenchymal cells	−3.173144	0.1336789	[52,53]
DAW1	Upregulated	Unclear	Unclear	2.507415	0.1336789	
MYBPC1	Upregulated	Unclear	Correlates with bone mineral density, likely by affecting skeletal muscle loss	3.601125	0.1841973	[54]
NPIPA8	Upregulated	Unclear	Unclear	4.324186	0.1867315	
NEFM	Downregulated	Inflammatory	Downregulation due to DNA methylation - Macrophage and CD8+ T cell infiltration in malignancy	−2.917603	0.2224579	[55]

**Table 4:** Top DEGs in subchondral bone of MetS-associated CTA, corrected for age, sex, and CRP.

Genes	Regulation	Likely Effect of Gene	Pathophysiological Description	Log2FoldChange	−log10 (FDR)	References
TGM2	Downregulated	Anabolic	Chondrocyte maturation and mineralized scaffold formation, Wnt/β-catenin signalling activation - Migration, mineralization, and osteogenic differentiation of mesenchymal cells	−3.3507315	0.0025472	[56,57]
KIF5A	Downregulated	Unclear	Unclear	−4.2775322	0.0611450	
BRSK1	Downregulated	Unclear	Unclear	−3.3693750	0.0775459	
PTPMT1	Upregulated	Anabolic	Increased mitochondrial metabolic capacity - Osteoblast differentiation from mesenchymal cells	1.0628621	0.2995526	[58,59]
VSNL1	Downregulated	Unclear	Unclear			
OPTN	Upregulated	Anabolic	NF-κB signalling suppression in rheumatoid arthritis - Inhibition of osteoclast differentiation via RANKL expression regulation - Decreased catabolic factor expression (MMP3)	−4.1565301	0.3016345	[58]
RALB	Upregulated	Unclear	Unclear	1.2887951	0.3016345	
MAST4	Downregulated	Anabolic (in osteogenesis) Catabolic (in chondrogenesis)	TGF-β1-dependent inhibition → increased SOX9 stability - Increased chondrogenic differentiation Increased β-catenin nuclear localization and Runx2 activity - Increased osteogenic differentiation	−1.9906119	0.3016345	[60]
KLHL3	Downregulated	Unclear	Unclear	−2.2875423	0.3018224	
ZC3H13	Downregulated	Inflammatory	m6A methylation - Inflammation and OA synovitis	−0.7312717	0.3018224	[61]
RALA	Upregulated	Anabolic	Increased SOX9 and ACAN protein level - Increased chondrogenic differentiation	1.4128225	0.3216342	[62]
DDIT4	Downregulated	Anabolic	Regulation of PGC1α levels and mitochondrial biogenesis - Enhanced chondrocyte survival under oxidative stress REDD1/TXNIP complex formation and suppression of mTOR signalling - Normal autophagy activation in chondrocyte	−2.6283417	0.3216342	[63,64]
MSANTD3	Upregulated	Unclear	Unclear	1.8272517	0.3216342	
AC005670.3	Downregulated	Unclear	Unclear	−1.1064848	0.3216342	
DNER	Downregulated	Anabolic Inflammatory	Activation of Notch signalling - Chondrocyte proliferation Modulation of immune response	−5.1965309	0.6513570	[65,66]
CD320	Downregulated	Anabolic	Increased HGF expression → activation of ERK 1/2 signalling - Bone marrow angiogenesis	−2.4803583	0.6513570	[67]
MYC	Downregulated	Anabolic	Increased chondrocyte proliferation and reduced cartilage degradation Increased ALP and BMP2 expression in ankylosing spondylitis - Increased fibroblast osteogenesis	−1.8769580	0.6513570	[68,69]
AC004057.1	Upregulated	Unclear	Unclear	2.9485435	0.6513570	
MX1	Upregulated	Catabolic Inflammatory	Regulation of chemokine release - Chondrocyte degeneration and cartilage degradation	1.6875061	0.6513570	[70]
NSMCE3	Upregulated	Anabolic	Interaction with SMC5/6 complex - DNA repair and cell cycle progression	1.0260843	0.6513570	[71]

**Table 5:** Top DEGs in subchondral bone of MetS-associated primary OA, corrected for age, sex, and CRP.

Genes	Regulation	Likely Effect of Gene	Pathophysiological Description	Log2FoldChange	−log10 (FDR)	References
SNORC	Upregulated	Inflammatory	Knockdown in IL-1β-induced chondrocytes → suppression of PI3K and JNK signalling - Decreased production of inflammatory factors (NO, IL-6, TNF-α, PGE2) and catabolic factors (ADAMTS5, MMP-13)	4.230058	0.0000012	[72]
SCRG1	Upregulated	Anabolic Inflammatory	Suppression of mesenchymal stem cell proliferation and induction of cartilage formation Inflammation and immune infiltration in OA synovitis through unknown mechanisms	3.418468	0.0000034	[73]
MTARC1	Downregulated	Anti-inflammatory	Reduced NO production - Protective against non-alcoholic fatty liver disease	−1.235527	0.0000066	[74]
NOTUM	Upregulated	Anti-inflammatory	WNT signalling inhibition and highly expressed in OA	4.631073	0.0000330	[75]
VAT1L	Upregulated	Inflammatory	Associated with plasma adiponectin level, which has contradictory effect - Activation of AMPK and NF-κB signalling → inflammatory/catabolic factor (IL-6, MMP-1, MMP-3) production Suppression of TNF-α production → anti-inflammatory (via M2 > M1 macrophage differentiation) effect	2.034787	0.0001065	[76,77,78,79,80,81]
CLU	Upregulated	Anabolic Anti-inflammatory	Inhibition of PI3K/Akt signalling - Suppression of IL-1β-mediated inflammation Inhibition of NF-κB signalling, respectively - Reduced MMP-13 production	3.397077	0.0001272	[82]
ACAN	Upregulated	Anabolic	Aggrecan is a key component of cartilage extracellular matrix	4.245406	0.0001343	[83]
STC2	Upregulated	Anabolic	Activation of ERK1/2 signalling in mesenchymal stem cells - Decreased adipogenesis - Increased osteogenesis	3.653243	0.0001470	[84]
SHISA4	Upregulated	Unclear	Unclear	2.167296	0.0001470	
FIBIN	Upregulated	Inflammatory	Inhibition → mitochondrial dysfunction in sarcopenia	3.109228	0.0001846	[85]
EPS8L2	Upregulated	Unclear	Unclear	2.131447	0.0001846	
FGFBP2	Upregulated	Inflammatory	Component of FGF signalling pathway - Inflammation in modic changes in human intervertebral disc	3.549677	0.0002803	[86]
ITIH6	Upregulated	Unclear	Unclear	5.202700	0.0003075	
H19	Upregulated	Catabolic	Regulation of miR-140-5p pathway - Increased MMP-1 and -13 expression - Chondrocyte apoptosis	3.035993	0.0003636	[87]
MIA	Upregulated	Catabolic	Inhibition of ERK 1/2 signalling - Reduced osteogenic differentiation - Increased aggrecan expression	3.796352	0.0003721	[88,89]
PANX3	Upregulated	Catabolic Inflammatory	Inhibition of WNT/β-catenin signalling - Increased Cx43 expression Inhibition of ERK 1/2 signalling - Increased MMP-13 expression	4.118535	0.0003854	[90,91]
SCX	Upregulated	Catabolic	Suppression of tension-induced osteoblast differentiation in periodontal ligament cells	1.833260	0.0004293	[92]
TRPV4	Upregulated	Anabolic	Activation of AMPK signalling and suppression of NF-κB signalling - Stress-induced osteogenic differentiation - Cartilage protection	2.907016	0.0006412	[93,94]
B3GNT9	Upregulated	Unclear	Unclear	1.733289	0.0009621	
SLC38A3	Upregulated	Unclear	Unclear	3.328527	0.0010543	

**Table 6:** Top N = 50 Reactome pathways implicated in MetS-associated primary OA.

Reactome Pathway	setSize	pANOVA	s.dist	p.adjustANOVA
Eukaryotic translation elongation	93	4.09 × 10^−29^	0.671	6.03 × 10^−26^
Peptide chain elongation	88	3.13 × 10^−27^	0.666	1.74 × 10^−24^
Viral mRNA translation	88	3.54 × 10^−27^	0.665	1.74 × 10^−24^
Selenocysteine synthesis	92	3.09 × 10^−25^	0.625	6.51 × 10^−23^
Eukaryotic translation termination	92	1.79 × 10^−24^	0.615	2.64 × 10^−22^
Unwinding of DNA	12	2.28 × 10^−4^	−0.614	2.65 × 10^−3^
Formation of a pool of free 40S subunits	100	3.23 × 10^−26^	0.612	7.93 × 10^−24^
Collagen biosynthesis and modifying enzymes	62	1.03 × 10^−16^	0.609	7.20 × 10^−15^
CS/DS degradation	11	4.92 × 10^−4^	0.607	5.18 × 10^−3^
SARS-CoV-1 modulates host translation machinery	36	4.56 × 10^−10^	0.600	1.68 × 10^−8^
Response of EIF2AK4 (GCN2) to amino acid deficiency	100	4.42 × 10^−25^	0.598	7.87 × 10^−23^
Nonsense-mediated decay (NMD) independent of the Exon Junction Complex (EJC)	94	1.73 × 10^−23^	0.596	1.96 × 10^−21^
SRP-dependent cotranslational protein targeting to membrane	111	1.23 × 10^−26^	0.586	4.54 × 10^−24^
Interleukin-15 signalling	14	1.48 × 10^−4^	−0.586	1.93 × 10^−3^
Interleukin-2 signalling	11	7.88 × 10^−4^	−0.584	7.68 × 10^−3^
Keratan sulphate degradation	11	8.05 × 10^−4^	0.583	7.68 × 10^−3^
Assembly of collagen fibrils and other multimeric structures	51	7.73 × 10^−13^	0.580	3.56 × 10^−11^
RIP-mediated NFkB activation via ZBP1	17	4.39 × 10^−5^	−0.572	6.88 × 10^−4^
Collagen chain trimerization	40	4.78 × 10^−10^	0.569	1.72 × 10^−8^
GTP hydrolysis and joining of the 60S ribosomal subunit	111	4.81 × 10^−25^	0.567	7.87 × 10^−23^
L13a-mediated translational silencing of ceruloplasmin expression	110	4.75 × 10^−24^	0.558	5.83 × 10^−22^
Collagen formation	82	5.42 × 10^−18^	0.552	4.18 × 10^−16^
Selenoamino acid metabolism	114	4.38 × 10^−24^	0.548	5.83 × 10^−22^
Polo-like kinase-mediated events	16	2.00 × 10^−4^	−0.537	2.38 × 10^−3^
HDMs demethylate histones	21	2.20 × 10^−5^	−0.535	3.90 × 10^−4^
FOXO-mediated transcription of cell death genes	16	2.67 × 10^−4^	−0.526	3.01 × 10^−3^
Cap-dependent translation initiation	118	9.28 × 10^−23^	0.523	9.11 × 10^−21^
Eukaryotic translation initiation	118	9.28 × 10^−23^	0.523	9.11 × 10^−21^
Diseases associated with glycosaminoglycan metabolism	36	1.13 × 10^−7^	0.511	3.26 × 10^−6^
CD22-mediated BCR regulation	12	2.88 × 10^−3^	−0.497	1.97 × 10^−2^
Formation of the ternary complex and, subsequently, the 43S complex	51	9.87 × 10^−10^	0.495	3.46 × 10^−8^
ZBP1(DAI)-mediated induction of type I IFNs	20	1.85 × 10^−4^	−0.483	2.27 × 10^−3^
Scavenging by Class A Receptors	16	8.81 × 10^−4^	0.480	8.27 × 10^−3^
Synthesis of Leukotrienes (LTs) and Eoxins (EXs)	17	7.34 × 10^−4^	−0.473	7.31 × 10^−3^
STAT5 activation downstream of FLT3 ITD mutants	10	9.65 × 10^−3^	−0.473	5.06 × 10^−2^
SARS-CoV-2 modulates host translation machinery	49	1.36 × 10^−8^	0.469	4.35 × 10^−7^
ECM proteoglycans	50	1.04 × 10^−8^	0.468	3.39 × 10^−7^
Regulation of IFNA/IFNB signalling	12	5.23 × 10^−3^	−0.466	3.18 × 10^−2^
Growth hormone receptor signalling	20	3.48 × 10^−4^	−0.462	3.71 × 10^−3^
GP1b-IX-V activation signalling	10	1.18 × 10^−2^	−0.460	5.84 × 10^−2^
Activation of the pre-replicative complex	32	7.42 × 10^−6^	−0.458	1.44 × 10^−4^
Transcription of E2F targets under negative control by DREAM complex	19	5.65 × 10^−4^	−0.457	5.82 × 10^−3^
Inhibition of replication initiation of damaged DNA by RB1/E2F1	13	4.93 × 10^−3^	−0.450	3.04 × 10^−2^
G0 and early G1	27	5.25 × 10^−5^	−0.450	8.06 × 10^−4^
Heme biosynthesis	13	5.13 × 10^−3^	−0.448	3.13 × 10^−2^
Dissolution of fibrin clot	12	7.30 × 10^−3^	0.447	4.12 × 10^−2^
Interleukin-3, Interleukin-5, and GM-CSF signalling	40	9.98 × 10^−7^	−0.447	2.41 × 10^−5^
Ribosomal scanning and start codon recognition	58	4.30 × 10^−9^	0.446	1.44 × 10^−7^
Regulation of FOXO transcriptional activity by acetylation	10	1.48 × 10^−2^	−0.445	6.78 × 10^−2^
Nonsense-mediated decay (NMD) enhanced by the Exon Junction Complex (EJC)	114	2.45 × 10^−16^	0.444	1.57 × 10^−14^

## Data Availability

The data presented in this study are available on request from the corresponding author.

## Abbreviations

AMPK	Adenosine monophosphate-activated protein kinase
BMP-2	Bone morphogenetic protein-2
CS	Chondroitin sulphate
C21orf62.AS1	Chromosome 21 Open Reading Frame 62 antisense RNA 1
cDNA	Complimentary Deoxyribonucleic acid
Cx43	Connexin 43
CTA	Cuff-tear arthropathy
DS	Dermatan sulphate
DKK1	Dickkopf-1
*DEGs*	Differentially expressed genes
FRZB	Frizzled motif associated with bone development
FOSB	FosB Proto-Oncogene
INTS6.AS1	Integrator complex subunit 6 antisense RNA 1
KNDC1	Kinase Non-Catalytic C-Lobe Domain Containing 1
Panx3	Pannexin 3
MIA	Melanoma inhibitory activity
MAPK	Mitogen-activated protein kinase
OA	Osteoarthritis
CD-RAP	Retinoic acid-sensitive protein
SIRT-1	Sirtuin-1
STC2	Stanniocalcin-2
TGF-β3	Transforming growth factor β3
VAT1L	Vesicle Amine Transport 1-Like
